# Impact of obstetric physiotherapy and transcutaneous electrical nerve stimulation (TENS) on pain management and gastrointestinal function following cesarean birth: A randomized controlled trial

**DOI:** 10.18332/ejm/191740

**Published:** 2024-09-05

**Authors:** Anna Pilch, Małgorzata Jekiełek, Beata Stach, Joanna Zyznawska, Marek Klimek

**Affiliations:** 1 Institute of Physiotherapy, Faculty of Health Sciences, Jagiellonian University Medical College, Krakow, Poland; 2 Institute of Nursing and Midwifery, Faculty of Health Sciences, Jagiellonian University Medical College, Krakow, Poland

**Keywords:** cesarean birth, postpartum care, pain management, transcutaneous electrical nerve stimulation, physiotherapy, gastrointestinal function

## Abstract

**INTRODUCTION:**

The study aimed to compare the impact of the physiotherapeutic method combined with TENS and physiotherapy alone on post-cesarean pain levels and the time required for intestinal peristalsis recovery. The study was conducted at the Specialist Hospital in Kraków, from January to March 2020.

**METHODS:**

The study was designed as a parallel randomized controlled trial (RCT). Participants were randomly assigned to one of three groups: TENS (n=52), nTENS (n=50) and control group (n=34), based on block randomization of 6. The allocation sequence was provided using a computer-generated random list. The participants were 136 postpartum primiparous women after cesarean birth, aged ≥18 years, having a healthy newborn, with no contradictions to TENS. The TENS group received a physiotherapeutic procedure involving a 20-minute exercise program plus a 40-minute session of TENS. The nTENS group received physiotherapeutic procedure alone, and the control group was under the routine care of midwives. The pain was assessed using the Numerical Rating Scale (NRS) at 6, 7, 12 and 24 hours after cesarean birth and twice during verticalization.

**RESULTS:**

TENS and nTENS groups had decreased pain intensity immediately after the intervention compared to the control group (p=0.002, p=0.027, respectively). During the first stage of the verticalization, the smallest increase in pain was observed in the TENS (p=0.044 compared to nTENS, p=0.000 compared to the control group). Within the increase in the pain score, the intestinal peristalsis recovery time was longer. In both groups undergoing physical therapy, a shortened recovery time of intestinal peristalsis was demonstrated (p=0.000).

**CONCLUSIONS:**

The proposed physiotherapy program, combined with TENS and instruction, proved effective in relieving post-cesarean pain and accelerating the time to first defecation and should be considered part of the standard patient management program in maternity units.

## INTRODUCTION

Cesarean birth, which is estimated to reach a global rate of almost 30% by 2030, equivalent to to approximately 38 million cesarean deliveries performed annually, is the most frequently performed obstetric procedure and one of the most common surgical procedures among women worldwide^1^. Poland is among the European countries with the highest percentage of cesarean births (about 44.2% of all births)^2^. Due to the increased safety, the procedure is treated as an alternative to vaginal birth, even if there are no medical indications. Nevertheless, like any other surgical procedure, cesarean birth carries a certain risk of complications, affecting both newborns and mothers. In countries with a high standard of perinatal medicine, maternal mortality and morbidity following cesarean birth is 8 to 10 times higher compared to vaginal birth^3^. The average age of women having a first birth is also constantly increasing, and such patients require more comprehensive care to prevent perinatal complications, the risk of which increases with the age of the pregnant woman^2^. This poses a significant challenge for perinatal care professionals, including physiotherapists^4^.

Women after a cesarean section (CS) birth, compared to those after vaginal birth, face greater limitations regarding normal functioning, mainly related to mobility and hygiene^5^. This is due to perioperative complications and pain. The surgical incision penetrates several layers of tissue, which causes pain during the healing and regeneration process. Uterine contractions also contribute to the onset of pain^6^. Postoperative discomfort is further exacerbated by the presence of flatulence and intestinal constipation^7^. The body can regulate the sensation of pain by releasing inhibitory neurotransmitters that completely or partially block the transmission of pain impulses. Endogenous oxytocin can also have an inhibitory effect on neurotransmitters and can help reduce the pain experienced by a woman after birth and during lactation^8^. Despite a better understanding of pain mechanisms and the introduction of advanced, safe analgesics and anesthetic techniques, postoperative pain management in European countries remains an area that needs much improvement^9^. Ineffective postoperative pain management can have negative consequences for patients. Persistent acute postoperative pain delays post-surgery mobility, increasing the risk of complications, including pulmonary (hypoventilation, pulmonary infection), gastrointestinal (reduced motility) and muscular (muscle strength decrease)^10^.

Pain can also hinder the initiation of breastfeeding by blocking the release of oxytocin and prevent proper newborn care in the immediate postpartum period, which can adversely affect early mother–neonate interactions and bonding^8^.

Effective pain management does not only involve pharmaceuticals but is the result of coordinated perioperative care provided by a multidisciplinary team, which is facilitated by the implementation of enhanced recovery after surgery (ERAS) procedures. Such a solution minimizes potential complications and accelerates recovery after surgery, as well as reducing the length of hospitalization by up to 0.5–1.5 days compared to standard management, thereby reducing hospitalization costs^4,11^.

Physiotherapy should constitute an integral part of ERAS regardless of the type of birth^12,13^. Early introduction of proper exercise program reduces the risk of complications and significantly accelerates full recovery after birth^13,14^. Research on individual elements of obstetric physiotherapy confirms its importance in the recovery after childbirth, improving the physical condition and general well-being of the mother, eliminating pregnancy-related adaptive symptoms and the consequences of perinatal complications. The physiotherapeutic techniques used in the clinical postpartum period include early mobilization to prevent venous and lymphatic stasis, early introduction of cardiorespiratory exercises, teaching proper techniques of standing up and sitting down that limit the contractions of the rectus abdominis muscles, pelvic floor muscle training (PFMT), and teaching optimal positions for breastfeeding^14^. In addition to standard physiotherapeutic methods, physical methods can also be used, the most effective of which in post-cesarean birth patients in terms of analgesic properties is the transcutaneous electrical nerve stimulation (TENS)^15^.

Recent data suggest that TENS not only modulates pain perception by recruiting Aβ afferent fibers in the dorsal horns of the spinal cord, which prevents or hinders the activation of pain-conducting fibers but also reduces pain hypersensitivity in the tissues surrounding surgical incisions. Laboratory studies showed a partial reduction in primary hypersensitivity and a complete reduction in secondary hypersensitivity. In comparison, opioids such as fentanyl and morphine were not as effective in reducing pain hypersensitivity. In addition, TENS was found to reduce the release and expression of excitatory neurotransmitters (glutamate and substance P), activation of glial cells, cytokines and inflammatory mediators in the dorsal horn. Simultaneously, μ-opioid, serotonin and delta-opioid receptors involved in analgesia are stimulated. The analgesic effect of TENS also stems from the activation of GABAA and muscarinic receptors (M1, M3) in the spinal cord^16^.

It was demonstrated that TENS allows to reduce the intake of analgesic medications while keeping the pain level under control, which is a significant advantage in terms of health for the mother and allows to reduce hospitalization costs. In addition, it is also a promising method of pain relief in patients with a history of opioid drug abuse^17^. Conclusions from previous studies corroborate the effectiveness and complete safety of TENS in relieving pain in post-cesarean birth women immediately after stimulation^18-20^, as well as its analgesic effect during physical activity^16^.

In addition, the findings from a systematic review of the effects of electrostimulation treatment in accelerating the recovery of normal intestinal peristalsis in patients after abdominal surgery, point to a 57% effectiveness of TENS in this respect. The authors highlight the great potential of TENS, but also the need for further research in this area^21^.

Accelerating the recovery of gastrointestinal function remains a great challenge, as according to studies, from 5%^21^ to even 31,7%^22^ woman after cesarean birth experience mild symptoms of delayed intestinal peristalsis recovery, referred to as postoperative ileus (POI). POI prolongs hospitalization, causes bloating that exacerbates lower abdominal pain and hinders breastfeeding and proper neonatal care. Therefore, preventing POI and supporting healthy intestinal peristalsis recovery should be one of the priorities in the perioperative period. A 2018 systematic review of 18 publications on the effects of electrostimulation treatment in accelerating intestinal peristalsis recovery showed a 55% effectiveness of this method in patients after abdominal surgery (the effectiveness of TENS was assessed at 57%). The authors highlight the great potential of TENS in accelerating the recovery of proper intestinal function, but at the same time point to the need for further research in this area^23^.

Despite the evidence of the effectiveness and benefits of physiotherapy and electrostimulation in perinatal care, there are no guidelines in this respect in the standards of perinatal care organization in Poland^24^. The problem of the lack of educational, training and therapeutic programs in perinatal care has been also pointed out by other studies^14,25,26^.

The aim of this study is to evaluate the effect of selected physiotherapeutic methods on postoperative pain and intestinal peristalsis in the early postpartum period after cesarean birth.

## METHODS

### Trial design and randomization

**Figure 1:**
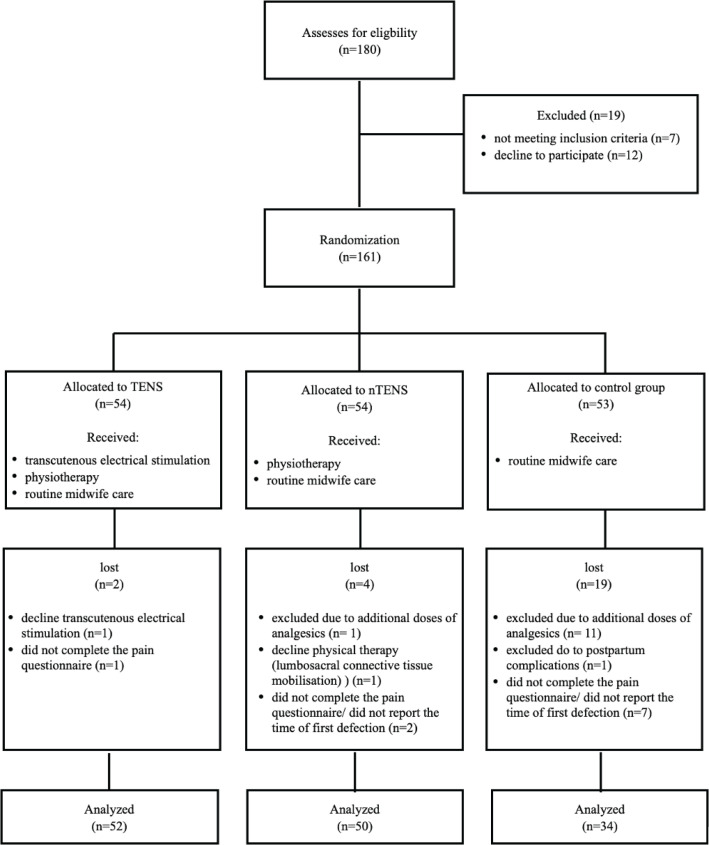
Flowchart of participants of the RCT at the Specialist Hospital in Kraków, Poland, January–March 2020

The present study was designed as a three-group, parallel randomized controlled trial (RCT) to compare the impact of the physiotherapeutic method combined with TENS, and physiotherapy treatment alone, on postoperative pain levels in post-cesarean birth women and on the time required for intestinal peristalsis recovery. Participants were assigned to one of three groups: TENS (n=52), nTENS (n=50) and control group (n=34) (Figure 1), based on block randomization of 6 to maintain a balanced number of participants in each treatment group, reducing variability, and avoiding selection bias. The random allocation sequence was provided by the statistician, using the random number generation computer method and subsequent sequences were given to the researcher in an envelope after evaluation of the participants for inclusion and exclusion criteria and collected preliminary data. Participants were not informed about group assignments; however, as the TENS device was applied only to the TENS group and the physiotherapy methods were not provided to the control group, participants could realize how they were assigned.

### Participants and setting

The study was conducted at the Specialist Hospital in Kraków, from January to March 2020 (ended with the beginning of the SARS-CoV-2 pandemic in Poland). The study was conducted on 136 women after elective cesarean birth. In all women, the Misgav-Ladach method under epidural and/or spinal anesthesia was performed. The same pain medication regimen was applied to all. By focusing on the principles of minimal intervention, the Misgav-Ladach method aims to provide a safer, more efficient alternative to traditional CS techniques^3^.

Inclusion criteria for the study were: written informed consent, aged ≥18 years, heaving a healthy newborn (Apgar score ≥8), and no contraindications for TENS (cardiac disease with a pacemaker or automated implanted cardiac defibrillator, skin irritation on TENS application sites). Multiparous women or with multiple pregnancies, with any postpartum complications (bleeding, hypertension) and women receiving additional doses of analgesics were excluded from the study.

Data were collected from the participants who met the inclusion criteria and confirmed to participate in the study in the patient rooms 6 and 7 hours after cesarean birth, during verticalization (between 7 and 8 hours). All participants were asked to complete the questionnaire regarding the level of pain after 12 and 24 hours after delivery and to record the time of first defecation.

### Interventions

The TENS group received a 40-minute TENS performed 6 hours after cesarean birth, using the MyoPlus 2 Pro, a dual-channel stimulator. Two adhesive gel electrodes with a diameter of 5 cm each were placed 3 cm above and below the incision line. Stimulation was performed at a frequency of 100 Hz, with a pulse duration of 100 μs and an individually selected intensity, defined as a distinct tingling sensation, with no or little muscle activity. The level of current intensity was increased, according to the tolerance threshold of the patient. Due to the lack of standard guidelines regarding the duration of TENS, the time was selected based on a literature review indicating the effective use of TENS postoperatively, including post-cesarean, for 30–60 minutes^18-22^. The electrostimulation was followed by a physiotherapeutic procedure involving a 20-minute exercise program consisting of breathing exercises combined with forced expiration exercises and wound care instruction, circulation-boosting exercises, pelvic tilt exercises, lumbosacral connective tissue mobilization, thoracic muscle stretching exercises, isometric exercises of the quadriceps thigh muscles and gluteal muscles. The nTENS group received the same physiotherapeutic procedure as the TENS group, and the control group was under the care of midwives, receiving routine care including the administration of medications, nursing activities, assisting with verticalization, instruction on newborn care and breastfeeding.

In addition, instruction was given verbally and, after the intervention, also in writing, in the form of a list of guidelines for the postpartum period. The instruction, in addition to the exercises performed under the supervision of the physiotherapist, included recommendations concerning methods of performing PFMT, correct micturition and defecation habits; posture-correcting exercises, optimal body positions during breastfeeding and nursing of the newborn; prevention of diastasis recti abdominis (DRA), scar mobilization, and return to normal physical activity.

### Outcomes

Using the eleven-point Numerical Rating Scale (NRS)^27^, the pain was assessed as follows: 6 and 7 hours after cesarean birth, twice during verticalization (between 7 and 8 hours after cesarean birth), which was classified into two stages: change of position from lying to sitting (Verticalization I) and from sitting to standing (Verticalization II). Women were also asked to complete the questionnaire regarding the level of pain after 12 and 24 hours after delivery (using the NRS) and report the time of first defecation.

### Statistical analysis

Statistical analysis was performed using STATISTICA Version 13.1 software. Values of tests and coefficients at the level of p<0.05 were assumed as statistically significant. Quantitative variables are presented as mean and standard deviation, and range. The normal distribution of variables was verified using the Shapiro-Wilk test. One-way analysis of variance (ANOVA) was performed to compare means across the study groups to determine whether the differences observed were statistically significant. Subsequently, Tukey’s test was performed to identify which pairs of groups exhibited statistically significant differences. The chi-squared test was used to determine whether there was a significant association between categorical variables, while Spearman’s rank correlation coefficient assessed the strength and direction of the association.

### Ethical considerations

Ethical approval was provided by the Bioethics Committee of Jagiellonian University (Protocol approval number: 1072.6120.137.2019, 27 June 2019). Written informed consent was obtained from all participants after hospital admission. This study was conducted as a randomized controlled trial (RCT) with random allocation of participants to intervention and control groups. However, it was not registered with a trial registry prior to participant enrollment. The authors acknowledge the importance of trial registration for transparency and ethical research conduct. All other methodological and ethical standards for conducting an RCT were strictly followed.

## RESULTS

The study was conducted in a group of 161 post-cesarean birth women, aged 19–41 years (mean=32.5, SD=4.9), divided into the groups: TENS (n=54), nTENS (n=54) and control (n=53). In all, 25 participants were lost or excluded after randomization, and 136 remained and were analyzed. Loses after randomization with reasons are provided on the flowchart (Figure 1). Descriptive characteristics are presented in Table 1.

**Table 1:** Anthropometric data of the participants of the RCT at the Specialist Hospital in Kraków, January– March 2020 (N=136)

	*TENS (N=52) Mean ± SD (Range)*	*nTENS (N=50) Mean ± SD (Range)*	*Control (N=34) Mean ± SD (Range)*
**Age** (years)	32.0 ± 5.2 (19–40)	33.0 ± 5.4 (19–41)	33.0 ± 5.1 (19–41)
**Height** (cm)	167.3 ± 4.8 (153–178)	166.7 ± 16.7 (154–178)	167.4 ± 5.3 (155–178)
**Body mass** (kg)	75.0 ± 6.3 (59–89)	75.0 ± 9.1 (59–91)	75.9 ± 6.5 (62–92)
**BMI** (kg/m^2^)	26.8 ± 2.0 (22.3–32.0)	27.0 ± 3.2 (21.8–34.0)	27.0 ± 1.7 (24.4–31.2)

BMI: body mass index.

### Post-operative pain

Following ANOVA testing, no significant differences among group means were observed in resting pain levels after 6 hours after cesarean birth (p=0.889), while after 7 hours following the birth, pain levels between groups were significantly different (p=0.007). Subsequently, Tukey’s test was conducted to identify which pairs of groups exhibited statistically significant differences. The analysis revealed significance between TENS and control group (p=0.005). After 12 and 24 hours, Tukey’s test identified statistically significant differences in both pairs: TENS-control group (p=0.027, p=0.000 after 12 and 24 hours, respectively) and nTENS-control group (p=0.018, p=0.000). For none of the resting pain measurements were there any significant differences observed between TENS and nTENS (Table 2).

**Table 2:** Pain intensity (on the NRS) after cesarean birth in TENS, nTENS and control group in the RCT at the Specialist Hospital in Kraków, January–March 2020 (N=136)

*Rest pain*	*TENS (N=52)*	*nTENS (N=50)*	*Control (N=34)*	*p*		
	*Mean ± SD*	*Mean ± SD*	*Mean ± SD*	*TENS-control*	*nTENS-control*	*TENS-nTENS*
6 h after CB	3.94 ± 1.13	4.04 ± 1.11	3.94 ± 1.21	1.000	0.920	0.902
7 h after CB	1.94 ± 1.09	2.24 ± 1.21	2.76 ± 1.21	0.005	0.109	0.402
7 h vs 6 h after CB	2.00 ± 1.22	1.80 ± 1.01	1.18 ± 0.90	0.002	0.027	0.615
12 h after CB	3.48 ± 0.98	3.44 ± 1.07	4.09 ± 1.14	0.027	0.018	0.979
24 h after CB	2.00 ± 0.82	2.04 ± 0.78	3.29 ± 1.09	0.000	0.000	0.971
**Pain during mobilization**						
Verticalization I	4.23 ± 1.02	4.70 ± 0.97	5.29 ± 0.91	0.000	0.019	0.044
Verticalization II	3.27 ± 1.26	3.32 ± 1.12	4.44 ± 0.99	0.000	0.000	0.973

CB: cesarean birth.

Percentage distribution of pain levels at 6 hours after cesarean birth, showed the highest percentage of the patients rating pain as moderate, in each group. In turn, after 7 hours, the majority of the patients rated the pain as mild. After 12 hours, the median pain level increased again; however, in more than half of the subjects in TENS and nTENS, it continued to be mild, while in the control group, it remained at a moderate level. The greatest differences between the TENS and nTENS groups and the control group could be observed 24 hours following cesarean birth (Table 3). The change in the experienced pain level between measurements at 7 and 6 hours following the birth was also compared, as a physiotherapeutic intervention in TENS and nTENS was undertaken at that time. Following ANOVA testing, significant differences among group means were observed (p=0.002), Subsequently, Tukey’s test revealed significance between TENS and the control group (p=0.002) and between nTENS and the control group (p=0.027).

**Table 3:** Percentage distribution of pain scores on the NRS at 6, 7, 12 and 24 hours after cesarean birth in the RCT at the Specialist Hospital in Kraków, January–March 2020 (N=136)

*Pain NRS*	*6 h*			*7 h*			*12 h*			*24 h*		
	*TENS %*	*nTENS %*	*Control %*	*TENS %*	*nTENS %*	*Control %*	*TENS %*	*nTENS %*	*Control %*	*TENS %*	*nTENS %*	*Control %*
0 No pain	0.0	0.0	0.0	7.7	4.0	0.0	0.0	0.0	0.0	0.0	0.0	0.0
1–3 Mild pain	36.6	34.0	44.2	86.6	80.0	76.5	53.8	60.0	35.3	96.2	98.0	58.8
4–6 Moderate pain	59.7	62.0	55.8	5.7	16.0	23.5	44.3	38.0	61.8	3.8	2.0	41.1
7–8 Severe pain	3.8	4.0	0.0	0.0	0.0	0.0	1.9	2.0	2.9	0.0	0.0	0.0

**Figure 2:**
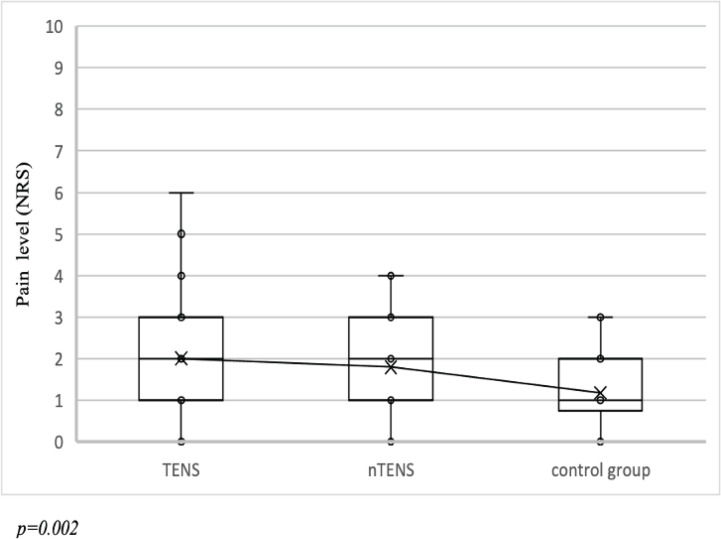
The difference in pain intensity between 7 and 6 hours after cesarean birth between TENS (n=52), nTENS (n=50) and the control group (n=34) in the RCT at the Specialist Hospital in Kraków, Poland, January–March 2020

The highest reduction in pain intensity was observed in TENS (by 6 points), while in the control group the pain level decreased by a maximum of 3 points (Figure 2).

Women were also asked twice to assess their pain level during verticalization, which was classified into two stages: change of position from lying to sitting (Verticalization I) and from sitting to standing (Verticalization II). During both stages, the lowest pain levels were declared by patients in TENS, whereas the highest value was recorded in the control group. ANOVA test showed significant differences among groups during both stages of verticalization (p=0.000). Subsequently, Tukey’s test revealed significance during Verticalization I between all pairs: TENS-nTENS (p=0.044), TENS-control group (p=0.000), nTENS-control group (p=0.019). However, during Verticalization II, no significance was revealed between groups with physiotherapy methods applied (p=0.973) (Table 2).

The chi-squared test identified no significant association between BMI and the intensity of postoperative pain (in subsequent measurements, after 6, 7, 12 and 24 hours: p=0.356, 0.478, 0.755, and 0.882).

### Intestinal peristalsis

**Figure 3:**
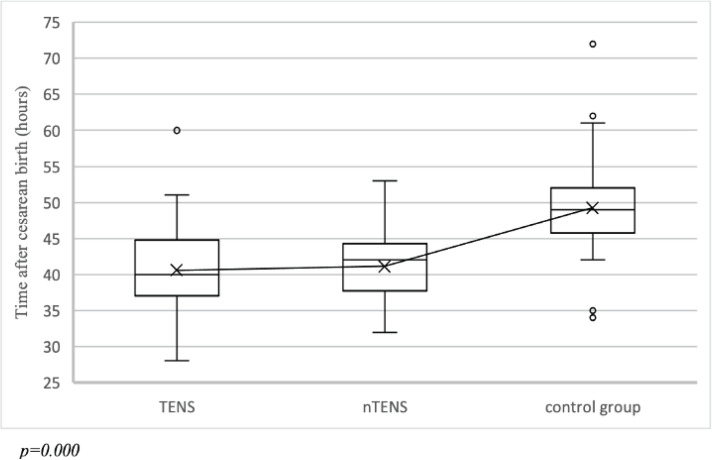
Time of first defecation after cesarean birth in TENS (n=52), nTENS (n=50) and the control group (n=34) in the RCT at the Specialist Hospital in Kraków, Poland, January–March 2020

The effect of the physiotherapeutic methods used on intestinal peristalsis recovery was assessed by the time of first defecation (in hours after cesarean birth). The shortest and longest time was observed in TENS and the control group, respectively (Figure 3). ANOVA test showed significant differences among groups (p=0.000).

The significant association between pain after 7, 12 and 24 hours after cesarean birth and time of intestinal peristalsis recovery was identified in the chi-squared test (p=0.000, p=0.022, p=0.000, respectively). Subsequently, Spearman’s rank correlation test revealed that within the increase in the pain score, the intestinal peristalsis recovery time was longer. However, the correlation was weak (p=0.008, ρ=0.228 after 7 hour; p=0.011, ρ=0.218 after 12 hours; p=0.002, ρ=0.338 after 24 hours).

The chi-squared test was used to identify the association between intestinal peristalsis recovery and the patients’ BMI, but no statistically significant results were obtained in this regard (p=0.183).

## DISCUSSION

The present study demonstrated the effectiveness of selected physiotherapeutic methods in relieving pain in patients. The pain declared by the women in the control group was significantly higher in all measurements compared to the groups where physiotherapy methods were introduced. The exercises under the supervision of a physiotherapist were combined with instruction. The focus was on breathing exercises performed with gentle pressure on the incision. It might have been these elements of the exercise program that had the most impact on reducing pain levels in the study groups. Calm, deep breathing is part of many relaxation techniques and is commonly incorporated into pain management strategies. It not only improves lung function, promotes blood circulation and healing, but can also induce a state of relaxation, resulting in reduced activation of the sympathetic nervous system^28^. The pain level may also have been influenced by connective tissue manipulation. Bauer et al.^29^ showed that the latter can reduce pain levels in post-operative patients by stimulating the secretion of β-endorphins. The effects of manual techniques on muscle relaxation, alleviating anxiety and improving satisfaction with care are also noteworthy^29^. Simonelli et al.^30^ conducted research on primiparous women after cesarean birth and found a positive correlation between connective tissue manipulation and reduction in pain levels (by 1.8 points), immediately after the intervention. In the present study, a similar observation was made, but this was due to the entire exercise program and instruction provided. Further research is needed to assess the effectiveness of its components.

The physiotherapeutic method that deserves particular attention is TENS. In the present study 40-minute TENS was performed. Similar effects of a 50-minute^18^ and even a 30-minute treatment^19^ were obtained by other authors. Mehendale and Revadkar^31^ demonstrated the efficacy of as little as 15-minute stimulation treatment when performed twice. According to Zimpel et al.^15^, the effects of TENS can last up to 24 hours after stimulation. In the present study, at 7 hours after cesarean birth, the average pain level in the TENS and nTENS differed by 0.3 points, while in subsequent measurements at 12 and 24 hours, the differences tended to be smaller. This could suggest that the analgesic effect of TENS stimulation was weaker over time and that the differences between the TENS and the control could no longer be due to the stimulation itself, but to the combination of this method with exercise and instruction. Women were advised to repeat the exercises several times on the day following cesarean birth, in addition to one session performed under the supervision of a physiotherapist. In a study by Burti et al.^32^, a single 45-minute exercise program, performed 8 hours postpartum, was shown to be effective in relieving pain. The exercise session involved breathing exercises, exercises preventing deep vein thrombosis, pelvic floor muscle exercises and abdominal massage. After intervention the pain in post-cesarean birth women improved by 32.23% compared to the initial pain level^32^.

Despite the fact that there is an increasing number of maternity hospitals in Poland equipped with electrostimulators (due to their increasing use in delivery wards), they are not used to relieve postpartum pain. To the best of the authors’ knowledge, there has been no study so far conducted in Poland on the use of TENS in post-cesarean birth pain. Based on a systematic review of complementary and alternative medicine (CAM) practices used in post-cesarean birth pain relief, only 10 studies had been published on the role of TENS in this area by 2020. However, the authors emphasize that further research on the use of electrostimulation in postpartum women is needed, due to the considerable heterogeneity of previous studies and the poor quality of some^15^.

The present study assessed the level of pain during verticalization and its correlation with the physiotherapy treatment received. When changing position from lying to sitting, the average pain level was significantly lower in TENS than in the other two groups, which indicates that the stimulation was effective in suppressing pain stimuli resulting from irritation of the surgical incision during the change of position.

According to studies, up to 100% of post-cesarean birth women report mobility limitations due to pain during sitting down and standing up, while 75% report pain during walking^33^. Similar studies have been conducted on patients after abdominal surgery and after hip fracture. They showed a reduction in pain during walking and deep breathing, an improvement in walking speed and distance covered in patients in the study group, compared to the control group^34^, as well as general improvement in patient mobility^35^. The authors suggest that the reduction of hyperalgesia after TENS may be more effective for pain experienced during activity than for resting pain^35^.

In the present study, TENS was found to be effective only in the first stage of verticalization. When changing position from sitting to standing, pain levels were comparable with the nTENS group. Patients in both groups, however, experienced significantly lower pain levels during verticalization than patients in the control group. This may point to greater effectiveness in terms of pain relief of breathing exercises during verticalization and instruction in proper wound care. This finding is supported by Weerasinghel et al.^14^ who showed that as little as 10 minutes of preoperative instruction was effective in terms of pain relief in the first 2 days after cesarean birth, during such activities as changing position in bed, standing and walking. In the present study, the association between pain after 7, 12 and 24 hours after cesarean birth and time of intestinal peristalsis recovery was identified. Within the increase of the pain score, the intestinal peristalsis recovery time was longer. Although the correlations were weak, they do indicate a trend. Considering that pain can additionally delay intestinal peristalsis, its effective relief is all the more important.

In our study, the average times of first defecation, reported by participants, in the TENS and nTENS were significantly shorter than in the control group: 40.6, 41.1 and 49.2 hours, respectively. In the study of Zhou et al.^7^, in the group that received electrostimulation, the average times to first defecation were much shorter, 28.9 versus 42.5 hours in the control group. These differences may be due to such factors as electrode placement and current frequency. Zhou et al.^7^ used TENS at a low, modulated frequency of 2–10 Hz and electrodes were placed at acupuncture points on the lower extremities. In contrast, in the present study high frequency was selected for relieving acute pain. Since these results, it is worth considering a combination of high-frequency TENS in the first hours after cesarean birth, with low-frequency TENS for subsequent stimulation or electroacupuncture stimulation.

Regarding no significant difference between the TENS and nTENS in the present study, the effect of electrostimulation should be considered. It can be suggested that the most important factor in terms of the reduction in the time to first defecation was the instruction and exercise program introduced. This effect was observed following TENS stimulation and an exercise program including pelvic exercises – repetitive backward flexion, which, due to the resulting gentle activation of the transversus abdominis muscle, indirectly stimulates intestinal peristalsis^12^. The role of connective tissue manipulation also deserves attention. Performed in the lumbosacral region, sacral region and sacroiliac joints were included in the physiotherapy program due to their role in relieving pain and visceral dysfunction by stimulating segmental and suprasegmental reflexes. This has been shown to be effective in patients with chronic obstructions, in terms of not only defecation frequency but also time of defecation^36^.

### Strengths and limitations

The strength of the study is that it was, to our knowledge, the first in Poland to include such a set of physiotherapeutic methods, including TENS in the early postpartum period after cesarean birth. There is a lack of research on nonpharmacological methods of pain relief and on the effect of physiotherapy on the functioning of women in the first days after cesarean birth, and our study was an attempt to fill this gap.

However, this study has some limitations to the generalizability of findings, including that it was conducted in a single center, the control group was quite small due to multiple losses to follow-up and data about intestine peristalsis were self-reported by the participant, which creates a risk of some inaccuracy. Moreover, in the present study, TENS and physiotherapy were applied only once. In subsequent studies, it would be advisable to evaluate the effect of multiple applications of this treatment. Further research is also needed on the effectiveness of the individual components of perinatal physiotherapy to develop a comprehensive management program to improve the quality of post-cesarean birth patient care.

## CONCLUSIONS

The proposed physiotherapy program, combined with instruction, proved effective in relieving resting pain and pain during verticalization in post-cesarean birth women. The exercises performed by the patients also accelerated the time to first defecation. TENS proved to be a useful measure, especially for alleviating pain during the first stage of verticalization. The use of the physiotherapy methods in question should be considered part of the standard patient management program in maternity wards. Further research is needed to develop comprehensive guidelines to improve the quality of post-cesarean birth patient care.

## Data Availability

The data supporting this research are available from the authors on reasonable request.
